# Ischemic Stroke Secondary to Left Atrial Papillary Fibroelastoma: A Report of a Rare Case

**DOI:** 10.7759/cureus.92357

**Published:** 2025-09-15

**Authors:** Muhammad Y Nawaz, Arsalaan Sayyed, Mohammad Khan, Mishal K Siddiqui, James J Cappola, Aliyaan Chaudhry

**Affiliations:** 1 Internal Medicine, Campbell University School of Osteopathic Medicine, Lillington, USA; 2 Orthopedic Surgery, Campbell University School of Osteopathic Medicine, Lillington, USA; 3 Pediatrics, Campbell University School of Osteopathic Medicine, Lillington, USA; 4 Biological Sciences, University of Maryland, College Park, College Park, USA

**Keywords:** cardiac tumor, echocardiography, embolization, ischemic stroke, papillary fibroelastoma

## Abstract

Cardiac papillary fibroelastoma (PFE) is a rare benign cardiac tumor that typically arises on valvular endocardium and carries the potential for serious complications despite its non-malignant nature. We present a case of an elderly patient with a PFE in an unusual location, leading to stroke. The patient’s history, diagnostic workup, surgical management, and outcomes are detailed. This case is noteworthy due to the tumor’s atypical left atrial location, an exceedingly rare site, and its presentation as an acute ischemic stroke. In the context of this case, we discuss the clinical significance of PFEs, including their epidemiology, pathology, differential diagnosis, and management strategies. This case underlines the need for vigilance in detecting cardiac sources of emboli in stroke patients and supports early surgical excision of PFEs when feasible to avert potentially life-threatening sequelae.

## Introduction

Primary cardiac tumors are extremely uncommon, with an incidence in autopsy studies of well under 0.1% [1]. Papillary fibroelastoma (PFE) is among the most frequently encountered benign cardiac tumors in adults, second only to cardiac myxomas [2]. These tumors are small, frond-like endocardial growths that classically appear as a “sea anemone” on echocardiography due to their mobile, papillary projections​ [3]. PFEs are histologically benign lesions composed of a collagenous and elastic fiber core covered by endothelium​ [1]. Despite their benign nature, PFEs are clinically important because they can cause serious embolic complications. Described by multiple studies as a cause of myocardial infarction without coronary atherosclerosis, PFEs have since been implicated in a range of cardiovascular emergencies, including stroke, transient ischemic attacks (TIAs), acute myocardial infarction, peripheral emboli, and even sudden cardiac death [1-4].

PFEs most commonly arise from valvular endocardium, particularly on the aortic and mitral valves [1]. In two large series, approximately 55% of patients with PFE were male, with a mean age of around 60 years at detection​ [1]. Valvular involvement is predominant (~80-90% of cases), with the aortic valve being the most frequently affected site (reported in roughly 30-60% of cases across studies)​ [1]. The mitral valve is the next most common location, followed by the tricuspid and pulmonic valves, while non-valvular locations (such as the atrial endocardium) are exceedingly rare [5]. Indeed, PFE originating from the atrial free wall or atrial appendage comprises <7% of all cases, making the present case unusual and noteworthy [5]. In a large summed analysis of 725 patients, it is reported that PFEs have most commonly occurred on left-sided valves, particularly the aortic valve with 44% involvement and the mitral valve in 35% of cases [6]. Likewise, in a 162-patient series, it was discovered that over 80% of PFEs were valvular, most commonly involving the aortic valve [3].

Clinically, many PFEs are asymptomatic and discovered incidentally on imaging or at autopsy​ [1]. However, when symptoms do occur, they are often the result of embolization of tumor fragments or thrombi formed on the tumor’s surface. Stroke is the most common presentation of symptomatic PFE, reported in roughly 30% of patients in some series [1]. Other presentations include TIA, myocardial infarction from coronary artery embolism, syncope, or heart failure if the tumor causes valvular obstruction​ [1,4]. The risk of embolic events is substantial - one analysis found that 26 of 26 patients with surgically confirmed PFEs had symptoms attributable to embolization, and even among patients managed conservatively with presumed PFEs, embolic events occurred in about 6-7% over two years [6]. Given these risks, the clinical significance of identifying a PFE lies in preventing life-threatening complications.

Advances in imaging technology, particularly the widespread use of high-resolution transthoracic and transesophageal echocardiography (TEE), have led to more frequent recognition of PFEs in living patients [2]. This improved detection has revealed that what was once thought to be an exceedingly rare autopsy finding is likely underdiagnosed clinically. Nevertheless, management of PFE remains controversial in certain scenarios, balancing the “benign” pathology against the non-trivial risk of embolization. We herein report a case of a PFE in an atypical location (left atrium) that presented as an acute ischemic stroke. The case underscores the importance of considering cardiac tumors in the differential diagnosis of cryptogenic stroke. We also review the literature to contextualize this case, compare it with previously reported cases and series, and discuss the approach to management, including surgical excision versus medical therapy.

## Case presentation

**Figure 1:**
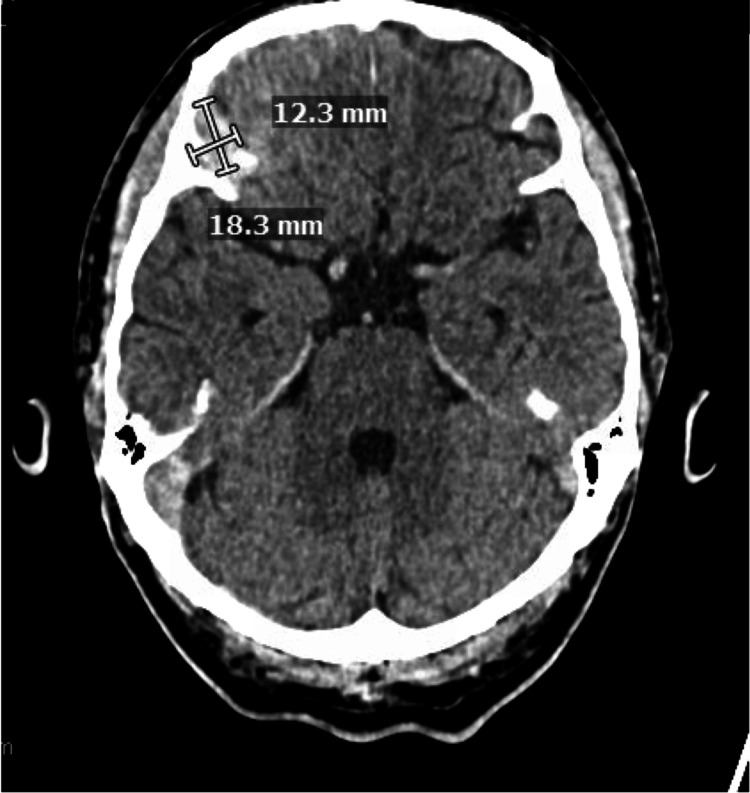
Acute right anterolateral frontal subdural hematoma with minimal mass effect. Additionally, there is a subacute chronic subdural hematoma more superiorly along the right frontal lobe.

A 54-year-old male patient with a significant past medical history of type II diabetes mellitus, coronary artery disease with prior myocardial infarction and stent placement, chronic heart failure (preserved ejection fraction), atrial flutter with rapid ventricular response, long COVID-19 syndrome, and prior pulmonary embolism (PE) (with inferior vena cava (IVC) filter placement), presented to the emergency department on January 13, 2025, complaining of progressive dizziness, generalized weakness, and recurrent episodes of syncope after a mechanical fall approximately three weeks prior. On admission, laboratory findings revealed hyperglycemia, hyponatremia, elevated blood urea nitrogen (BUN), and elevated creatinine (Table 1). Computed tomography (CT) head demonstrated two areas suggestive of subdural hematoma (SDH), and a CT angiogram (CTA) raised suspicion for a small vascular abnormality, later deemed an artifact on neurovascular consultation. Magnetic resonance imaging (MRI) of the brain revealed a 4-mm-thick subacute right frontal SDH and a small subarachnoid hemorrhage (SAH) (Figure 1).

**Table 1:** Laboratory findings on admission.

Laboratory Test	Result	Reference Range	Interpretation
Blood glucose	830 mg/dL	70-110 mg/dL (fasting)	Marked hyperglycemia
Sodium (Na⁺)	126 mmol/L	135-145 mmol/L	Hyponatremia
Blood urea nitrogen (BUN)	35 mg/dL	7-20 mg/dL	Elevated
Creatinine	2.0 mg/dL	0.6-1.3 mg/dL	Elevated

**Figure 2:**
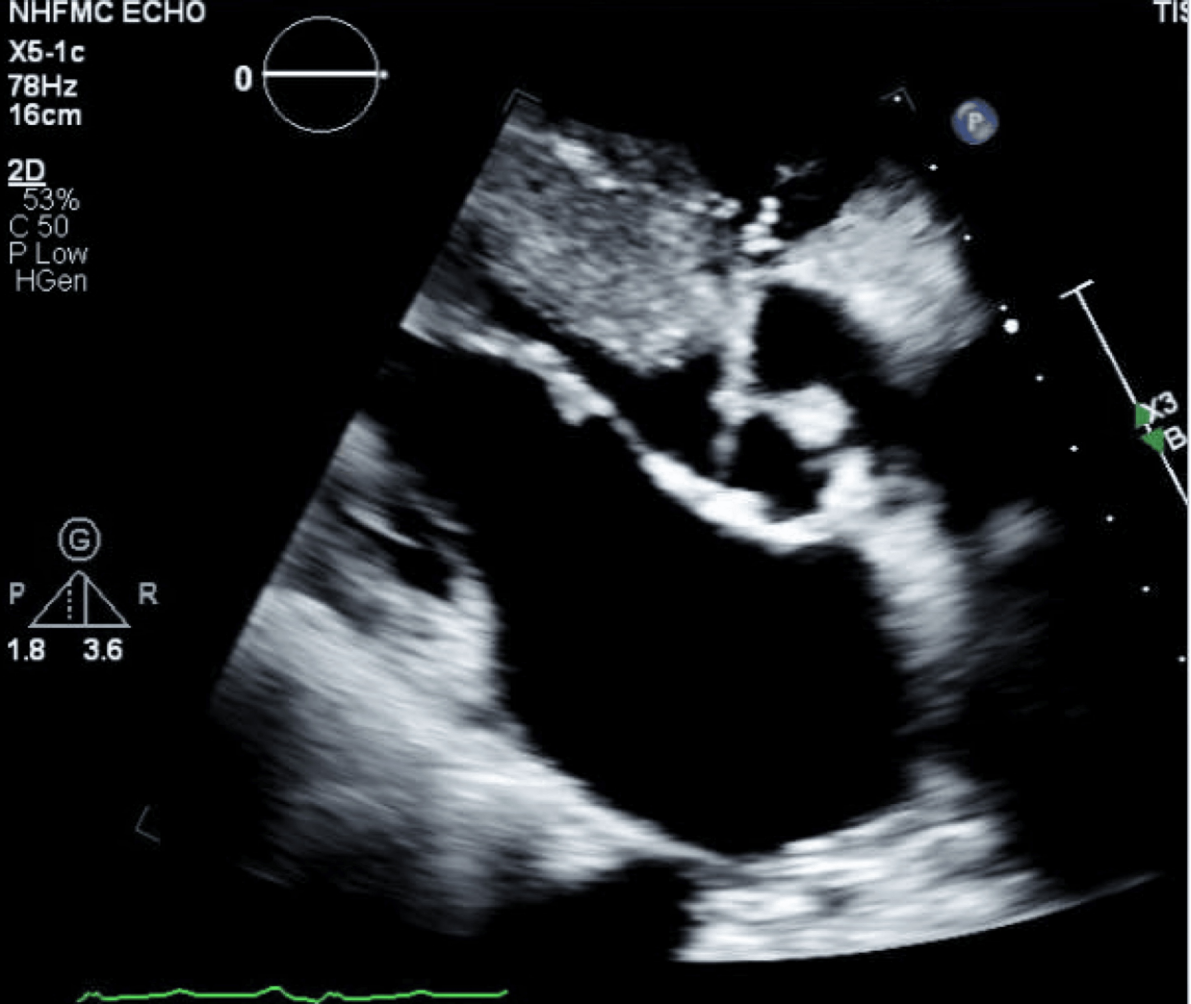
Transthoracic echocardiogram revealing a highly mobile, pedunculated mass attached to the aortic valve, consistent with a fibroelastoma. The lesion is obstructing the left ventricular outflow tract and displays a frond-like configuration.

During hospitalization, transthoracic echocardiography (TTE) showed an incidental finding of an independently mobile pedunculated mass measuring 0.6 cm × 0.9 cm on the noncoronary cusp of the aortic valve, consistent with a PFE. TEE confirmed the presence and location of the fibroelastoma (Figure 2). Given the patient's recent traumatic SAH and hematoma, immediate surgical intervention was deferred due to the high risk of worsening intracranial hemorrhage. Conservative management with medical stabilization was undertaken, and the patient was scheduled for outpatient cardiac surgical evaluation six weeks post-discharge. Cardiothoracic surgical consultation determined the necessity of cardiac catheterization before the planned future surgical resection due to the patient's known coronary artery disease history and prior myocardial infarctions treated with coronary stents. The patient stabilized neurologically with seizure prophylaxis (levetiracetam) and glycemic control. At discharge, plans were made for outpatient follow-up, cardiac catheterization to exclude concurrent coronary disease, and elective surgical evaluation for potential fibroelastoma excision.

## Discussion

This case presents a complex management of brain injury and an effective diagnosis of PFE in a medically fragile adult with significant cardiovascular comorbidities. PFE pathogenesis is suggested to arise from endothelial endocardial cells, though there is no clear etiology for the affliction, the most common site of PFE is the aortic valve, and the most common site of trauma is cerebral arteries [1,7,8]. The variety of preexisting conditions makes evaluating this patient's medical background particularly complex and allows for a more nuanced understanding of the relationships between PFE and these conditions. Fibroelastoma manifests alongside such conditions as TIA or stroke, angina, myocardial infarction, syncope, PE, blindness, peripheral emboli, and renal infarction [1-7].

The findings of this study are similar to other previously published case studies, one in which a 54-year-old female patient presented with central chest pain, alongside a history of hypertension without pre-existing cardiovascular complications, contrasting with the mechanical fall of our 54-year-old male patient, as well as his history of cardiovascular and pulmonary concerns. The female patient was diagnosed with non-obstructing coronary artery disease, paralleling the 54-year-old male patient’s pre-existing diagnosis [9]. Both patients' transesophageal echocardiograms showed mobile masses on the aortic valve (Figure 2) [9]. One striking difference between these cases is the absence of emboli in the female patient’s thorax, abdomen, and pelvis; in contrast, our patient has a history of PE and possible prevalence of peripheral emboli [9]. The diagnosis made for the 54-year-old female patient was an acute myocardial infarction, with the prognosis being the elevated high-sensitivity troponin I (hsTn-I) levels as well as ischemic conditions [9]. Remarkably, our patient was also found to have mildly elevated troponin levels measured at 35 ng/L with the presence of pre-diagnosed myocardial infarction; however, treatments differed due to our patient's operational risk in surgery. The 54-year-old female patient's case highlights the importance of early surgical excision of PFEs when possible to prevent embolization [10]. Risk for embolization remains substantial across pooled data. In a large pooled analysis, embolic events occurred in 30% of the time with respect to symptomatic patients [6]. According to a retrospective and prospective study, one in four patients experienced cerebrovascular accidents, often a stroke [3].

Our patients' greatly elevated creatinine and BUN levels (azotemia) upon admission indicate the potential for the development of a renal condition. Though there is no notable patient history of renal dysfunction, there is a significant link between pre-diagnosed PE and acute kidney injury (AKI) [11]. Our patient's levels of 2.0 mg/dL elevated creatinine, 35 mg/dL elevated BUN, and 126 mmol/L reduced Na are indicators of AKI. PE is known to be a unique cause of heart failure, which our patient suffers from, and given the patient's underlying factors, it is likely that chronic heart failure has contributed to the development of cardiorenal syndrome [11,12]. There are numerous factors, such as hemodynamic status, hypoxia, and hypoperfusion, which can lead to the development of AKI [11]. Peripheral emboli from cardiac PFE are known to have caused renal infarction, especially occlusion of the coronary arteries, but tumor fragments have been histologically identified in pulmonary arteries (in cases of PE) [7]. Though creatinine and BUN have traditionally been used to diagnose AKI, some research may suggest that a more sensitive biomarker, such as cystatin C found in human serum, which is comparable in mechanism to the release of troponin due to injury in myocardial cells, may allow for a more precise diagnosis of AKI [13]. Urinalysis results for the patient upon admission were negative, implying that there was no sign of oliguria in the patient, which indicates a lower likelihood of AKI [14]. Our patient's AKI may have been transient, as one day after admission, creatinine and BUN levels returned within normal range.

There may be a need for the patient to undergo cystatin C testing to rule out AKI moving forward in their treatment plan, as cystatin C is a greater measure of glomerular filtration rate (GFR). AKI carries significant morbidity risk as well as a need for long-term dialysis and increased risk for cardiovascular events, necessitating risk management [13-15].

Another notable report from our 54-year-old patient was of right leg weakness following the pre-admission diagnosis of PE. Right leg weakness may be a potential indicator of past or present deep vein thrombosis (DVT), considering that the patient has had an IVC placement to prevent thrombi from entering pulmonary arteries from the lower body. The complex of DVT and PE is referred to as venous thromboembolism [16]. The patient's history of oxygen use before and after admission further strengthens the presence of current PE. Our patient has had a history of long-term COVID-19, which is a significant risk factor for the development of DVT as well as PE, with longer-term COVID-19 causing the most increase in risk for PE after six months [17]. Given that our patient's PFE is found on the left aortic valve, research suggests that systemic embolization is not out of the picture, with embolization of the coronary arteries, lungs, brain, kidneys, and lower limbs having been commonly reported to cause severe lasting damage [18].

Though the mechanical fall that preceded the patient's admission was listed as the trauma source of both the patient’s SDH and subdural arachnoid hemorrhage, the presence of PFE raises the possibility of stroke and TIA due to its well-established research relationship with both. TIA and stroke in patients with PFE are known to be caused via the formation of a fibrin thrombus on the tumor, alternatively by the fragment of the tumor itself [4-19]. The presence of embolic thrombi or tumor fragments in the cerebral arteries is likely to cause cerebral ischemia, which is known to result in strokes [4-19]. PFEs are relatively soft and gelatinous tumors that have a friable texture conducive to fragmenting to provoke cerebrovascular stroke [19].

An individualized approach is considered during patient management, but high-risk patients often require surgery. Gowda et al. recommended surgical excision in all left-sided lesions, mobile PFEs, and in those who are asymptomatic. Similarly, Sun et al. reported that surgical intervention was correlated with favorable outcomes and diminished recurrence rates [3].

## Conclusions

While a definitive link between PFE and this patient's ischemic event cannot be causally established, taking into account his numerous comorbidities and hypercoagulable risk factors, PFE on the aortic valve remains a firmly established embolic source. This case underlines the importance of considering PFE in the differential diagnosis of cryptogenic stroke. Identification of this structural lesion leads to individualized management implications, including the potential role for surgical excision to prevent life-threatening embolic events.
